# The Inhibitory Effects of Nesfatin-1 in Ventromedial Hypothalamus on Gastric Function and Its Regulation by Nucleus Accumbens

**DOI:** 10.3389/fphys.2016.00634

**Published:** 2017-01-05

**Authors:** Shengli Gao, Feifei Guo, Xiangrong Sun, Nana Zhang, Yanling Gong, Luo Xu

**Affiliations:** ^1^Department of Pathophysiology, School of Basic Medicine, Qingdao UniversityQingdao, China; ^2^Department of Clinical Laboratory, The Affiliated Hospital of Qingdao UniversityQingdao, China; ^3^Department of Pharmacy, College of Chemical Engineering, Qingdao University of Science and TechnologyQingdao, China

**Keywords:** nesfatin-1, ventromedial hypothalamus, nucleus accumbens, gastric function, food intake

## Abstract

**Aim:** The aim of this study was to investigate the effect of nesfatin-1 signaling in the ventromedial hypothalamus (VMH) on gastric functions, as well as the regulation of these effects by nucleus accumbens (NAc) projections to VMH.

**Methods:** The expression of c-fos in nesfatinergic VMH neurons induced by gastric distension (GD) was measured using the double fluoro-immunohistochemical staining. The firing rates of neurons were monitored with single-unit extracellular electric discharge recording. The projection of nesfatinergic neurons from NAc to VMH was observed by fluorogold retrograde tracer combined with fluoro-immunohistochemical staining. The effect of nesfatin-1 in VMH or electric stimulation in NAc on gastric function was studied by measuring food intake, gastric acid output, gastric motility, and gastric emptying, and the ability of the melanocortin-3/4 receptor antagonist SHU9119 or the anti-nesfatin-1 antibody to block nesfatin-1 in the VMH was assessed.

**Results:** Expression of c-fos was observed in VMH nesfatinergic neurons following GD in rats. Further, nesfatin-1 delivery to single GD-responsive neurons changed the firing rates of these neurons in the VMH. In awake, behaving rats, intra-VMH administration of nesfatin-1 inhibited food intake, gastric acid output, gastric motility, and gastric emptying. These effects were abolished by SHU9119. Fluorogold retrograde tracing showed nesfatinergic neural projection from the NAc to the VMH. Electrical stimulation of NAc modified the firing rates of the VMH neurons and inhibited food intake and gastric functions. The pretreatment with an anti-nesfatin-1 antibody in the VMH reversed the effects of NAc electrical stimulation on the VMH neuronal firing rates and gastric function.

**Conclusions:** Nesfatin-1 in the VMH inhibited food intake, gastric acid output, gastric motility, and gastric emptying. A nesfatinergic pathway between NAc and VMH transmitted metabolism-regulating signals.

## Introduction

Obesity is widely regarded as an important public health problem throughout the developed world (Ng et al., 2014). Food intake is controlled by multiple neurochemical and endocrine signaling systems including several neuropeptides. Nesfatin-1, one of the few anorexigenic neuropeptides, has received much recent focus for its role in regulating food intake (Oh et al., 2006; Maejima et al., 2009). Nesfatin-1 is derived from the precursor nucleobindin-2 (NUCB2) and expressed in the nucleus accumbens (NAc), paraventricular nucleus (PVN), ventromedial hypothalamus (VMH), and arcuate nucleus (ARC), among other brain nuclei (Oh et al., 2006; Goebel et al., 2009). Experimentally, intracerebroventricular (i.c.v.) or intravenous (i.v.) injection of nesfatin-1 in rats decreases food intake and body weight via a leptin-independent, melanocortin-dependent signaling pathway (Oh et al., 2006; Maejima et al., 2009).

Earlier studies used i.c.v. administration of nesfatin-1, so the brain nucleus or nuclei responsible for these effects is not known. Further, the receptor for nesfatin-1 is yet unknown, making a mechanistic understanding of nesfatin-1 signaling in the central nervous system difficult to ascertain. To begin to answer these questions, we have injected nesfatin-1 in PVN and ARC (Li et al., 2013; Guo et al., 2015), and found that nesfatin-1 signaling in these areas has an inhibitory role in gastric motility. Moreover, these effects were caused by nesfatinergic projections from lateral hypothalamic area (LHA) to PVN or from PVN to ARC.

The VMH plays a key role in the regulation of feeding behaviors and the mediation of satiety (Duggan and Booth, 1986). In rats, VMH lesion enhances mRNA and protein expression of nesfatin-1 in white adipose tissues, stomach, and duodenum, as well as increases gastric emptying (Osaki et al., 2012; Tian et al., 2014). However, it is not known if the expression of nesfatin-1 in the VMH is affected by feeding and if injection of nesfatin-1 in the VMH modifies gastric function. Based on the anorexigenic role of nesfatin-1 and the satiety signals from VMH to the gastrointestinal tract, our hypothesis is that nesfatin-1 might be a “satiety” transmitter in the VMH and might play an important role in inhibiting gastric motility, emptying, and acid secretion.

Beside the direct regulation of the digestive system, the brain also influences food intake through reward mechanisms that are necessary for the “wanting” or “liking” of food. In brain, the shell of NAc contributes to processes like incentive motivation and reward learning, essential in food consumption behavior (Michaelides et al., 2013; Resendez et al., 2013). Indeed, many studies have shown an interaction between the NAc shell and food intake (Will et al., 2007; van der Plasse et al., 2012; Castro and Berridge, 2014). Though nesfatin-1 is abundantly expressed in NAc, the role for nesfatin-1 in NAc is not clear. Here we examine the possible role for nesfatinergic projections from NAc in modulating hypothalamic metabolic centers.

In this study, we aim to better understand the effect of nesfatin-1 in the VMH and NAc on food intake, gastric motility, gastric emptying, and gastric acid output. Further, we identify a nesfatinergic connection between NAc and VMH and its possible role in regulating these effects.

## Materials and methods

### Animals

Adult Sprague-Dawley (SD) rats (protocol number: 0013219, Qingdao Institute for Drug Control, Shandong, China) weighing 250 ~ 300 g were housed under controlled illumination (12:12-h light-dark cycle starting at 8 a.m.) and temperature (25 ± 2°C) and had free access to laboratory chow pellets and tap water. Animal Guidelines were approved by the Qingdao University Animal Care and Use Committee.

### Immunofluorescence staining of VMH after gastric distension

Rats were divided randomly into 2 groups: control group (*n* = 4) and gastric distension (GD) group (*n* = 8). The GD was performed as described previously (Gong et al., 2013). All rats were fasted overnight and anesthetized with Inactin (100 mg/kg, i.p.; Sigma-Aldrich Chemical, USA). Following a midline cut on the abdomen, the fundus wall was incised and gastric contents were washed out with warm isotonic saline. A latex balloon (length: 3 cm, diameter: 3 cm, max volume: 12 ml) attached to a polyethylene catheter (PE-50) was inserted into the stomach via the incision and fixed by silk thread. The pylorus was ligated to prevent duodenal reflux into the stomach and change of gastric volume. The abdomen was then closed. The polyethylene catheter was connected to an electronic barostat (Distender Series II, G&J Electronics Inc, Canada). The balloon was dilated to a constant pressure of 60 mm Hg for 20 s with a 4-min inter stimulus interval for 2 h. The control group received no treatment.

Thirty minutes after GD, rats were perfusion-fixed with 4% paraformaldehyde. The brains were moved for post-fixing, dehydrating, embedding, and sectioning. The 15-μm slices through VMH were preincubated for 1 h with 0.01 M PBS (pH 7.4) containing 0.3% Triton X-100, and 5% goat serum, then were incubated overnight at 4°C with primary antibodies: rabbit anti-c-Fos antibody (1:100, sc-8047, Santa Cruz Biotechnology, U.S.A.) and rabbit anti-nesfatin-1 antibody (1:200, H-003-22; Phoenix Pharmaceuticals Inc, U.S.A.). After rinsing, the sections were incubated with goat anti-mouse (1:500, Cy3-conjugated, 115-165-003, Jackson ImmunoResearch, U.S.A.) and goat anti-rabbit (1:50, FITC-conjugated, 111-095-003, Jackson ImmunoResearch, U.S.A.) fluorescent secondary antibody at room temperature for 90 min. After washing off unbound secondary antibodies with PBS, the sections were mounted in Citifluor (Citifluor, London, UK). Photographs of fluorophores were taken under a TCS SP8 two-photon laser scanning confocal microscope (Leica Microsystems AG, Wetzler, Germany). The mean percentage increase of c-Fos positive cells in the VMH was calculated as: (average c-Fos positive cells in stomach-distended rat—average c-Fos positive cells in control rat)/(average c-Fos positive cells in control rat) × 100%.

### Electrophysiological recording in the VMH after GD

Forty rats were prepared for GD following the protocol mentioned above, and the electrophysiological recording procedure was based on the previously described method (Li et al., 2013). The anesthetized rats were bound on a stereotaxic frame, and craniotomy was performed around the coordinates: −2.5 mm posterior to Bregma and 0.8 mm lateral. A four-barrel glass microelectrode (5–15 MΩ) was advanced in an increment of 10 μm with the aid of hydraulic micropositioner into the area of VMH (Bregma: P: −1.8 ~−3.2 mm, L (R): 0.5 ~ 1.0 mm, H: 8.5 ~ 9.2 mm) according to the rat brain atlas of Paxinos and Watson (Paxinos, 2007). One barrel was filled with 0.5 M sodium acetate and 2% Pontamine sky blue to record neural discharging, the other three barrels connected with multi-channel pressure injector (PM2000B; Micro Data Instrument Inc., NJ, USA) contained either: nesfatin-1 (Phoenix Pharmaceuticals, Burlingame, CA, USA), SHU9119 (an antagonist of melanocortin-3/4 receptor; Sigma-Aldrich Chemical, MO, USA), or normal saline (NS). The drugs (<1 nl) were superfused on the surface of neurons by a short pulse gas pressure (1500 ms, 5.0 ~ 15.0 psi).

Once the microelectrode was advanced into the VMH and a stable firing pattern was recorded, GD stimulus was conducted. The change of neuronal firing rate was calculated by the formula: 100% × (firing rate of neurons after treatment−firing rate of neurons before treatment)/(firing rate of neurons before treatment). If the mean firing frequency changed via gastric distension by at least 20% from the mean basal level, the neurons were divided into GD-responsive, GD-excitatory (GD-E), or GD-inhibitory (GD-I), and no-response subcategories. Spike data was recorded and processed using PowerLab data acquisition system (AD Instruments Pty Ltd, Australia).

To verify the correct location of the microelectrode, an iron deposit of pontamine sky blue was formed at the tip of the electrode after a direct current (10 μA, 20 min). Then the rats were perfused with 10% buffered Formalin solution and the brains were frozen. Coronal sections were cut through the regions of the hypothalamus, stained with neutral red, cleared with xyline, and coverslipped. All the recording and microinjection sites were verified under light microscope.

### Food intake and gastric function test after VMH injection of nesfatin-1

For direct VMH injections, a cannula was implanted. Fasted rats were anesthetized and fixed in the stereotaxic apparatus. After craniotomy, a stainless steel guide cannula (24 gauge, 1.5 mm distance, Plastics One, VA, USA) was implanted into the VMH (Bregma: P: −1.8 ~ −3.2 mm, L (R): 0.5 ~ 1.0 mm, H: 8.5 ~ 9.2 mm). After anchoring the cannula and sealing all skull openings with dental acrylic, a 26-gauge obturator was placed in the cannula. After the recovery period of 10 ~ 14 d, the drugs were injected with a needle (29-gauge) inserted into the cannula and connected to a syringe by a 10 cm piece of polyethylene tubing.

Food intake was calculated according to a previously published method (Chen et al., 2012). Thirty cannula-implanted rats were randomly divided into 3 groups: NS, low-dose nesfatin-1 (10 pmol) and high-dose nesfatin-1 (100 pmol) groups. Rats were fasted at 15:00 on the experiment day and administered NS or nesfatin-1 in the VMH 4 h later. Next, food was placed in the cage, and total food intake in the following 12 h was calculated by measuring the weight of food containers. The outputs were recorded by Data Acquisition software 51,800 (Feed-Drink Monitoring System Ver. 1.31, Ugo Basile, Italy).

The secretion of gastric acid induced by 2-deoxy-D-glucose was studied using an acute gastric fistula method described previously (Xia et al., 2012). Thirty rats with implanted VMH cannulas were randomly divided into 5 groups: NS, nesfatin-1 (100 pmol), 2- DG (200 mg/kg, sc) + NS, 2-DG + nesfatin-1, and 2-DG + nesftin-1 + SHU9119 (1 nmol) groups. Rats were fasted for 16 h and then anesthetized with urethane (1.5 g/kg ip). After incision at the abdominal midline, the esophagus was ligated at the gastroesophageal junction (to preserve the vagal trunks carefully). A double-lumen cannula was inserted into the stomach via the ligated pylorus. Basal acid secretion was established by collection of gastric outputs in 5 ml of perfusate every 15 min for 30 min. The gastric lumen was subsequently rinsed with 5 ml of saline every 15 min for 120 min after drugs were administered in the VMH or subcutaneously. The gastric rinse solution was then titrated to pH 7.0 with 100 mM sodium hydroxide using an automatic titrator (COM-2500; Hiranuma Sangyo Co Ltd, Japan) for titratable acidity.

To measure gastric motility, 30 rats with implanted cannulas were randomly divided into 5 groups: NS, nesfatin-1 (100 pmol), SHU9119 (1 nmol), nesfatin-1 + SHU9119, and nesfatin-1 (dorsomedial hypothalamic nucleus, DMN) group. Rats in the last group were implanted cannulas in ventral DMN as a specificity control for VMH. Gastric motility measurement was conducted as previously described (Guo et al., 2015). After rats fasted overnight and anesthetized with Inactin, laparotomy was performed to expose the stomach. For measurement of gastric motility, a strain gauge was sutured onto the serosa of the gastric antrum, 0.5 cm caudal from the pyloric ring. The lead wire of the strain gauge was finally fixed at the nape of the neck with a 2 ~ 3 cm outside end by extending through a subcutaneous tunnel. After recovery for 2 days, the rats were fasted overnight and recorded the gastric motility on a polygraph (3066–23; Chengdu Precision Instruments, Sichuan, China) by connecting the lead wires. The phase III-like contractions were defined as clustered potent contractions with amplitudes of more than 4 g. After achieving stable baseline recordings for at least 20 min, nesfatin-1 or SHU9119 was administered to the VMH. Each animal was recorded for 2 h on 2 different days. The changes in gastric motility were evaluated by the percentage motor index (%MI) of the motor activity in the antrum. Values of the %MI for a 5-min period in the antrum were calculated by 100% × (area under the manometric trace for each 5-min period after nesfatin-1 or vehicle injection)/(area under the manometric trace for the 5-min period immediately before nesfatin-1 or vehicle injection).

Gastric emptying was determined by the phenol red method with a non-nutrient viscous solution as described previously (Guo et al., 2015). Eighteen rats with implanted VMH cannulas were randomly divided into 3 groups: NS, nesfatin-1 (100 pmol), and nesfatin-1 + SHU9119 (1 nmol) groups. In the dark phase, fasted rats were stimulated with nesfatin-1 or the mixture of nesfatin-1 and SHU9119. Immediately, the animals were fed 1.5 mL of a non-nutrient viscous solution by gavage. Twenty minutes later, rats were sacrificed and the gastric contents were collected. Phenol red concentration was fitted to a standard curve.

### Fluorogold retrograde tracing

After anesthetized and mounted on a stereotaxic apparatus, 10 rats were injected 3% Fluorogold (0.2 μl, Sigma-Aldrich Chemical, MO, USA) into the VMH. After a 7-day recovery, the rats were perfusion-fixed with 4% paraformaldehyde. The brains were removed for post-fixing, dehydrating, embedding, and sectioned. Fifteen micrometers sections through NAc were incubated overnight at 4°C with rat anti-nesfatin-1 antibody. After washing, the sections were incubated with goat anti-rat fluorescent secondary antibody at room temperature for 90 min. After washing off unbound secondary antibodies with PBS, the sections were mounted in Citifluor (Citifluor, London, UK). The photographs of fluorophores were taken under a TCS SP8 two-photon laser scanning confocal microscope (Leica Microsystems AG, Wetzler, Germany).

### Electrophysiological recording in the VMH after electrical stimulation in NAc shell

For electrical stimulation, the electrodes were implanted in the NAc shell. Rats were anesthetized and subsequently mounted in a stereotaxic frame. Craniotomy was performed around the coordinates: 1.8 mm posterior to Bregma and 1.7 mm lateral. Electrodes (David Kopf Instruments, Tujunga, CA, USA) were placed in the NAc shell (bregma: P: 0.8 ~ 2.8 mm, L (R): 1.0 ~ 2.5 mm, H: 5.0 ~ 7.0 mm) and secured to the skull with dental cement. After 3-d recovery, the electrode was attached to the cable from a stimulator (Master-9, AMP Instruments LTD, Israel) to stimulate nucleus with a radiofrequency output of square-wave current impulses.

Eighty rats with implanted stimulating electrodes in NAc shell were prepared for GD and electrophysiological recording in the VMH following the process mentioned above. Once a neuron in the VMH was confirmed to be the GD-responsive cell, the direct current stimulation (20 μA, 0.5 ms and 50 Hz) was added to NAc shell without or with the pretreatment of anti-nesfatin-1 antibody. Spike data was recorded and processed using PowerLab data acquisition system.

### Food intake and gastric function test after electrical stimulation in NAc

The detailed approaches of electrode implantation, food intake, gastric acid output, gastric motility, and emptying had been described above. Here are the animal groups in every experiment.

In food intake test, 40 rats was randomly divided into 4 groups: control, low (20 μA), high (50 μA) current, and high current + nesfatin-1 antibody groups. During test, the rats were placed in the test chambers and given access to a bowl of pre-weighed chow. The animals were electrically stimulated in the test chamber for 1 h (from 8 to 9 p.m.), and food was weighed at 2, 4, and 6 h after stimulation. In the high current + nesfatin-1 antibody group, nesfatin-1 antibody was pretreated in the VMH before the electrical stimulation.

Forty rats, implanted with electrodes in NAc shell, were used to check the secretion of gastric acid and were randomly divided into 5 groups: basal control, 50 μA, 2-DG (200 mg/kg, sc), 2-DG + 50 μA, and 2-DG + 50 μA + nesfatin-1 antibody groups. Rats in the 2nd, 4th, and 5th groups were stimulated with 50 μA current in the NAc shell, and nesfatin-1 antibody was injected into the VMH of rats in the 5th group.

To record the gastric motility after the electrical stimulation in NAc shell, 30 rats were randomly divided into 3 groups: control, 50, and 50 μA + nesfatin-1 antibody groups. Rats of the 2nd and 3rd groups received the 50 μA stimulation in the NAc shell and SHU9119 was only given to rats of the 3rd group.

In the gastric emptying study, 30 rats were randomly divided into 3 groups: the control group, 50 μA group, and 50 μA + nesfatin-1 antibody groups (*n* = 10 in each group).

### Statistics analysis

Statistical analyses were processed with SPSS 15.0 statistics software (SPSS Inc, Chicago, IL, USA). The results are presented as the mean ± SEM. The Kolmogorov–Smirnov test was used to assess normal distribution. For data with a normal distribution, two-way repeated-measures ANOVA or Student's *t*-test was applied, while data that were not normally distributed were assessed for statistical significance using the Mann–Whitney *U*-test between two groups and Kruskal–Wallis test among more than two groups. A *p* < 0.05 was considered statistically significant.

## Results

### Effect of GD on the activation of nesfatin-1-expressing neurons in the VMH

**Figure 1:**
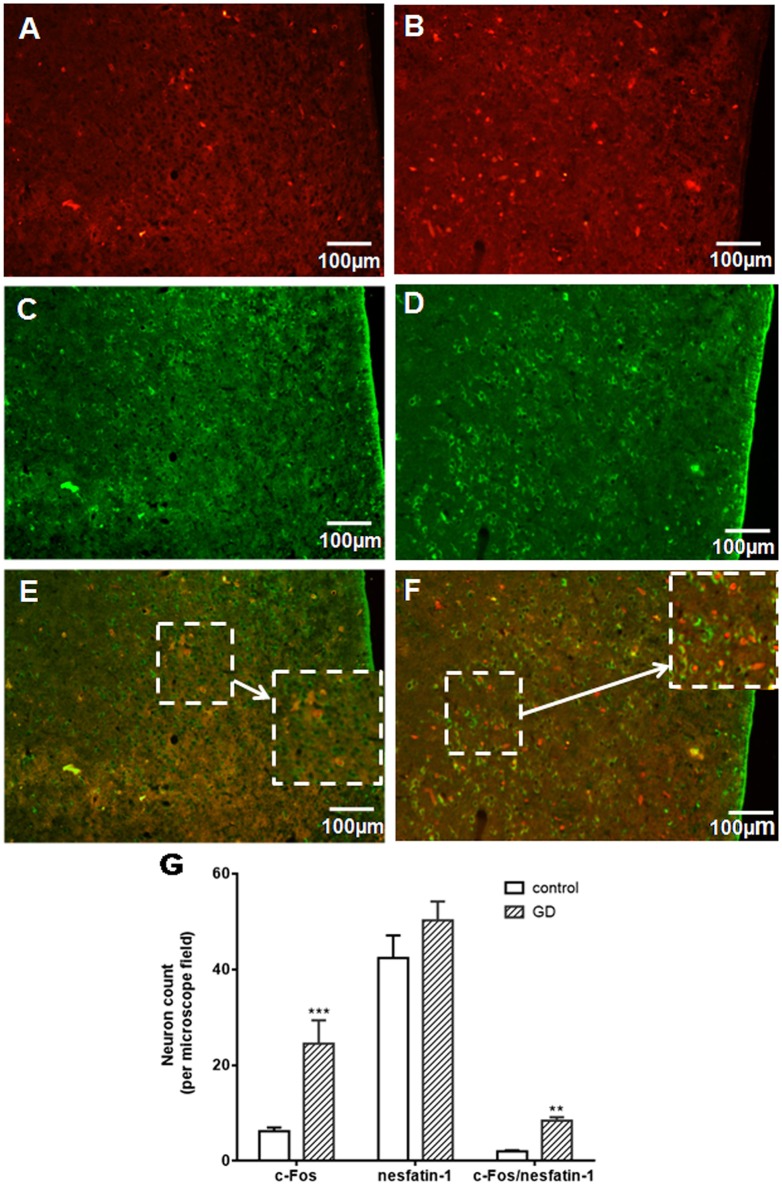
**Effect of gastric distension on c-fos expression in NUCB2/nesfatin-1 positive neurons of VMH. (A,B)** c-fos expression in the VMH in control **(A)** and rats following gastric distension **(B)**. **(C,D)** Nesfatin-1 expression in the VMH in control **(C)** and rats following gastric distension **(D)**. **(E,F)** Double immuno-detection of c-fos and NUCB2/nesfatin-1 in control **(E)** and rats following gastric distension **(F). (G)** Quantitative analysis of c-Fos, NUCB2/nesfatin-1, and co-expressing of c-Fos and NUCB2/nesfatin-1 in control and distended rats. ^**^P < 0.01 vs. control; ^***^P < 0.001 vs. control. White arrow points to neurons co-expressing NUCB2/nesfatin-1 and c-fos.

To assess activation of VMH nesfatinergic neurons following GD, double c-fos, and NUCB2/nesfatin-1 antibody labeling was performed. GD significantly increased the number of c-fos immunoreactive neurons in VMH to 24.5 ± 4.9 neurons per microscope field, higher than 6.2 ± 0.8 in control group (*P* < 0.001, Figures 1A,B,G). The results of the nesfatin-1 positive cell counting between normal and GD groups were not statistically significant (*P* > 0.05, Figures 1C,D,G). Among the c-fos immunoreactive neurons, some cells were double-labeled with the nesfatin-1 antibody (Figures 1E,F). 8.45 ± 0.72 double-labeled neurons per microscope field were observed in the GD group, whereas 2.01 ± 0.24 were observed in the control group (*P* < 0.05, Figure 1G). Thus, GD activated nesfatinergic neurons in VMH.

### Effect of nesfatin-1 on GD-responsive neurons in the VMH

In a pre-experiment, we tested the effects of different concentrations of nesfatin-1, including 0.1, 0.5, 1, 5, 10, 50, 100, and 200 nM, on the firing rate of VMH neurons. Our analysis of the dose-response revealed that the half maximal effective concentration (EC 50) of nesfatin-1 on firing rate is approximately 10 nM.

Out of a total of 88 VMH neurons in 40 rats, 64 (72.73%) were identified as GD-responsive neurons based on the change of firing rate elicited by GD. Among GD-responsive neurons, 42 (47.7%) showed an increase in firing rate following GD (GD-E), and 22 (25.0%) showed a decrease in firing rate following GD (GD-I).

**Figure 2:**
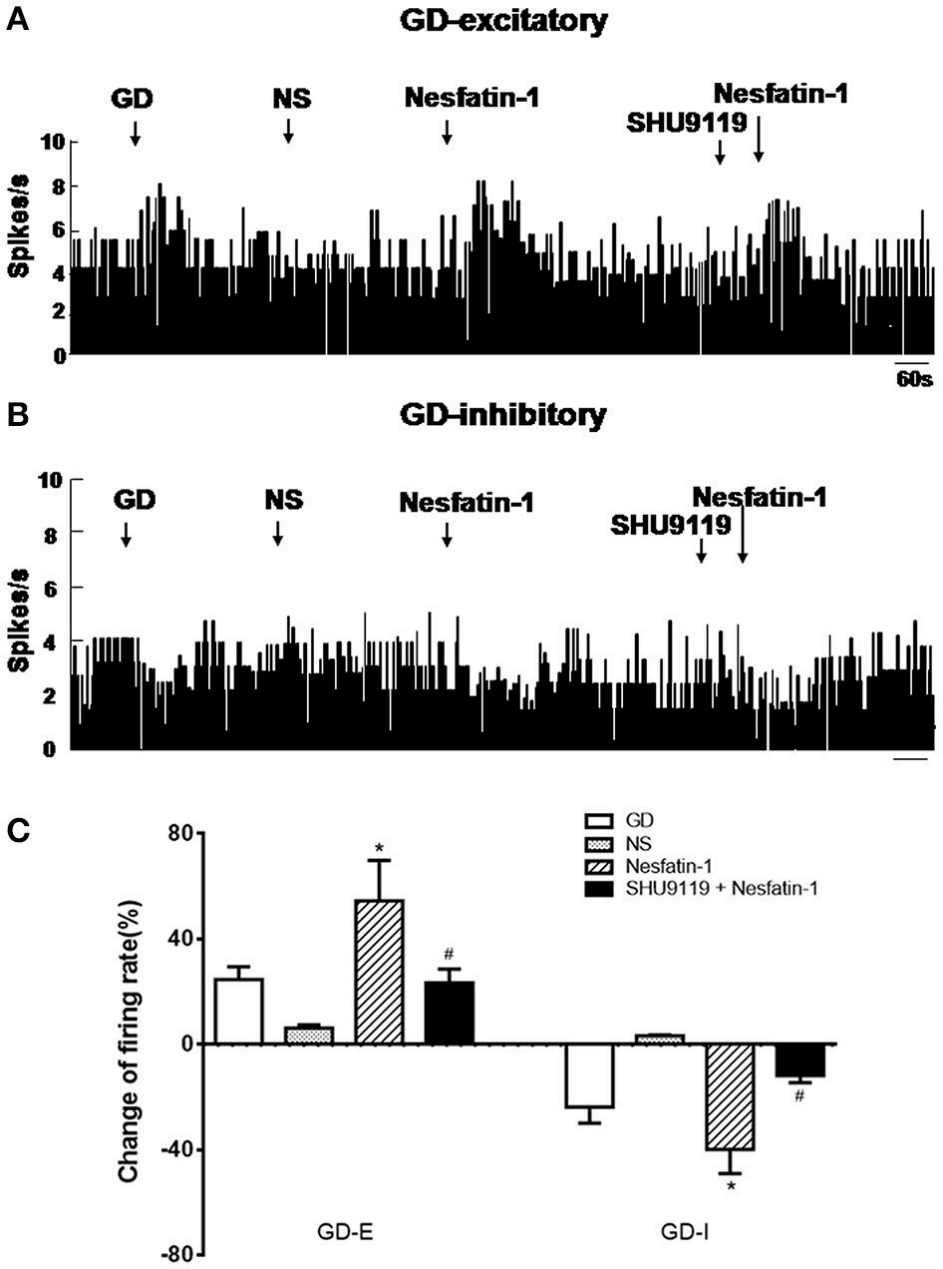
**Effect of nesfatin-1 on the firing rate of GD-responsive neurons in the VMH. (A)** GD-E neurons were mostly excited by nesfatin-1, **(B)** GD-I neurons were mostly inhibited by nesfatin-1, **(C)** shows the change in firing rate (%) of GD-responsive neurons in the VMH induced by nesfatin-1. Pre-treatment with melanocortin 3/4 receptors antagonist SHU9119 inhibited the effect of nesfatin-1. ^*^P < 0.05 vs. NS; ^#^*P* < 0.05 vs. nesfatin-1.

Next we assessed the effect of nesfatin-1 (10 nM, ~1 nL) via electrode ejection on the surface of the GD-responsive neuron. In 42 GD-E neurons, nesfatin-1 excited 61.9% (26/42) neurons, inhibited 16.7% (7/42), and did not affected 21.4% (9/42). Overall, nesfatin-1 increased the firing rate of GD-E neurons by 54.48 ± 15.39% (from 4.13 ± 1.21 to 6.38 ± 1.48 Hz, *P* < 0.05, Figures 2A,C). Sixteen of Twenty-two (72.7%) GD-I neurons were inhibited by nesfatin-1, resulting in a significant decrease in the firing rate from 3.84 ± 1.10 to 2.31 ± 0.77 Hz (39.84 ± 9.18%, *P* < 0.05, Figures 2B,C). Further, pretreatment with the melanocortin 3/4 receptor antagonist SHU9119 partially blocked the effect of nesfatin-1 on the GD-responsive neurons (Figure 2). SHU9119 alone had no effect on these neurons.

### Effect of nesfatin-1 microinjection in VMH on food intake and gastric function

**Figure 3:**
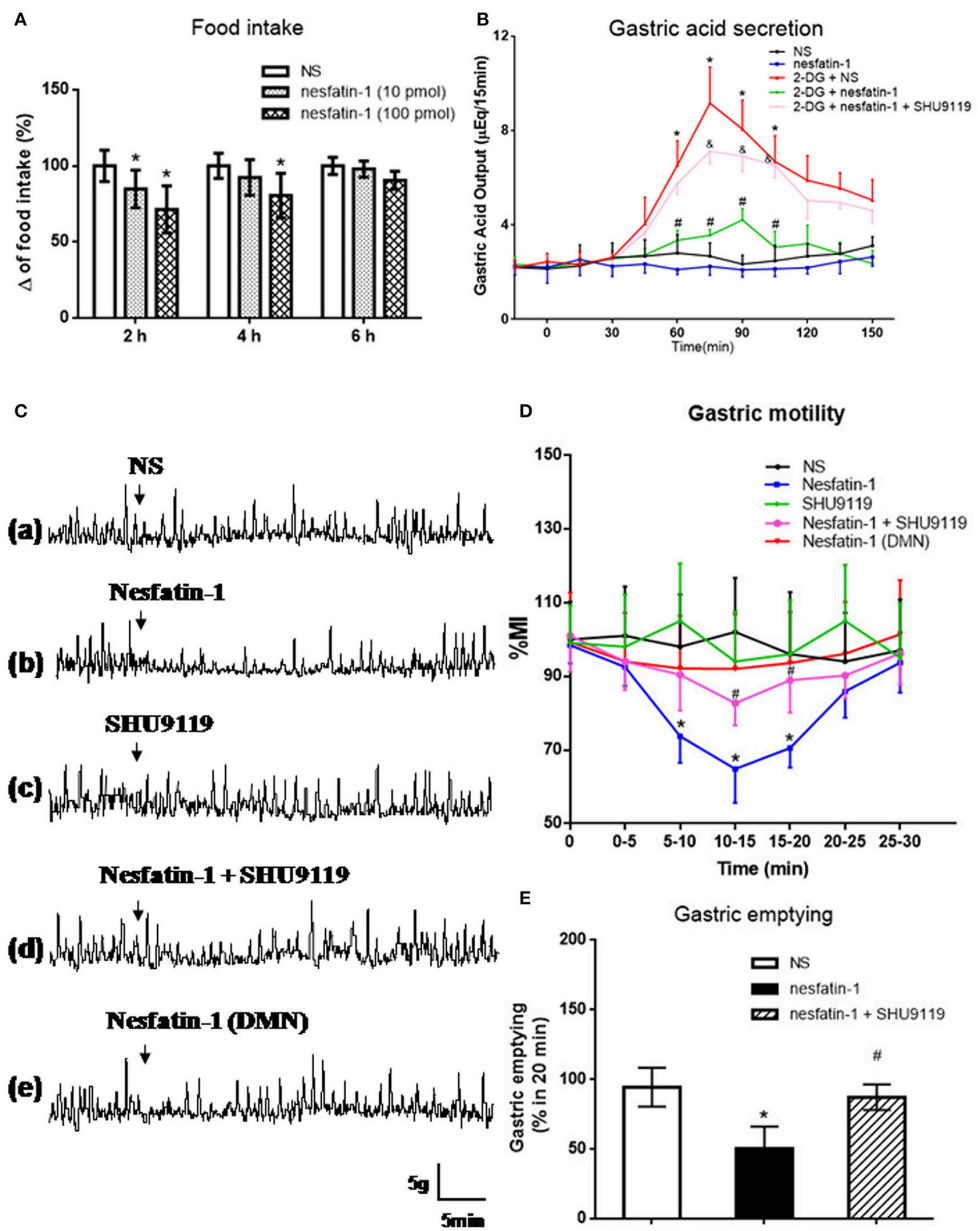
**Effect of VMH injection of nesfatin-1 on food intake and gastric function. (A)** VMH injection of nesfatin-1 decreased food intake during night period, ^*^P < 0.05 vs. NS. **(B)** Nesfatin-1 attenuated gastric acid secretion induced by 2-deoxy-D-glucose, ^*^P < 0.05 vs. NS, ^#^*P* < 0.05 vs. 2-DG + NS,^&^*P* < 0.05 vs. 2-DG + nesfatin-1; **(C,D)** Gastric motility was inhibited by nesfatin-1, ^*^P < 0.05 vs. NS, ^#^*P* < 0.05 vs. Nesfatin-1. **(E)** Nesfatin-1 delayed gastric emptying of a viscous non-caloric meal in conscious rats.^*^P < 0.05 vs. NS, ^#^*P* < 0.05 vs. nesfatin-1.

Nesfatin-1 was microinjected into the VMH of freely moving rats via cannula (Figure 3A). Low (10 pmol) and high (100 pmol) dose nesfatin-1 reduced cumulative food intake by 15.21 ± 2.34 and 28.63 ± 5.38%, respectively (*P* < 0.05, vs. NS group) over a 2 h period. Cumulative food intake over 4 h was reduced in the high dose group (*P* < 0.05, 80.47 ± 14.62%), but not in the low dose group, compared with the NS group. No statistically significant effect of nesfatin-1 on cumulative food intake was found 6 h after drug delivery, illustrating the acute nature of nesfatin-1's effect.

To assess the role for VMH nesfatin-1 in gastric function, we first examined gastric acid secretion. VMH injection of nesfatin-1 (100 pmol) had no effect on basal gastric acid secretion (Figure 3B). However, gastric acid output induced by 2-DG was inhibited by VMH injection of nesfatin-1 (Figure 3B). The peak acid production was decreased by 48.63 ± 10.21% (*P* < 0.05, vs. 2-DG group). In the 2-DG + nesfatin-1 + SHU9119 group, the gastric acid output was not different than the 2-DG alone group, suggesting that nesfatin-1's mechanism of action in the VMH involve the melanocortin-3/4 receptor.

Next, we examined gastric motility via an implanted strain gauge in freely moving rats. Rats receiving nesfatin-1 in VMH showed a change in percent motor index (MI%), a measure of gastric motility, starting 4.1 ± 0.8 min after nesfaitn-1 delivery with the peak change in MI% 15.2 ± 3.1 min following nesfatin-1. The MI% 10–15 min after nesfatin-1 injection was 35.2 ± 8.9% lower than that of NS group at the same time point [Figures 3Ca,b,D, *P* < 0.05]. Pre-treatment with 1 nmol SHU9119 blocked the effect of nesfatin-1 (*P* < 0.05, vs. nesfatin-1 group), while SHU9119 alone did not change gastric motility [Figures 3Cc,d,D]. To investigate the site specificity of VMH for these effects, we unilaterally injected nesfatin-1 into ventral DMN, a nucleus close to VMH. The results showed that there was no significant change for the gastric motility after nesfatin-1 was administrated into DMN [*P* > 0.05 vs. NS group, Figures 3Ce,D].

Lastly, we examined the effect of VMH nesfatin-1 on gastric emptying in awake rats. VMH injection of nesfatin-1 (100 pmol) inhibited the gastric emptying rate of a non-caloric viscous solution to 50.5 ± 15.6% [*P* < 0.05, vs. NS group (94.4 ± 13.9%), Figure 3E]. As with gastric acid secretion and gastric motility, pre-treatment of 1 nmol SHU9119 blocked the effect of nesfatin-1 on gastric emptying (87.2 ± 9.2%, *P* < 0.05, vs. nesfatin-1 group).

### Retrograde tracing of a nesfatin-1 pathway from NAc to VMH

**Figure 4:**
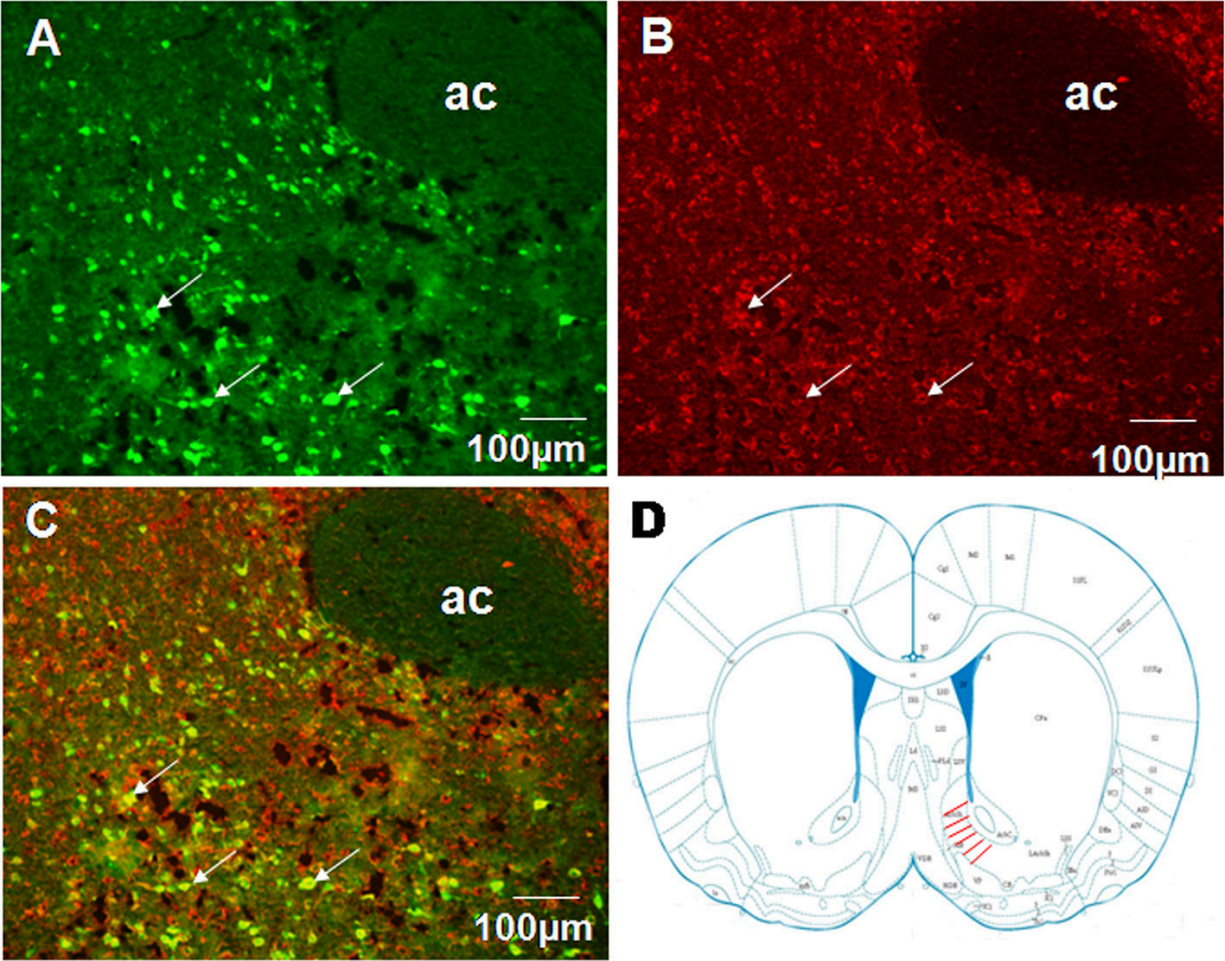
**Co-localization of retrograde labeling from VMH and NUCB2/nesfatin-1 in the neurons of the shell of NAc. (A)** The distribution of fluorogold retrotracing from the VMH to the NAc, white arrows indicate FG-labeled neurons, **(B)** The expression of NUCB2/nesfatin-1 in the NAc, white arrows indicate nesfatin-1positive neurons, **(C)** Double visualization of fluorogold and nesfatin-1 in NAc shell, white arrows indicate co-labeling of FG and nesfatin-1 in some neurons, **(D)** Schematic drawing illustrating the position of immunohistochemical figures in the NAc. Scale bars, 100 μm. Ac, core of NAc.

To examine projections to VMH we performed fluorogold retrograde labeling in VMH. 7 d after fluorogold injection, we observed abundant retrograde-labeled neurons in the shell of NAc (Figure 4A). In the same tissue sections, nesfatin-1 immunostaining revealed several nesfatin-positive neurons in the NAc shell (Figure 4B). Several flurogold/nesfatin-1 double positive neurons were observed in the NAc shell (Figure 4C). Quantitative analysis indicated approximately 43.2% fluorogold-labeled neurons expressed nesfatin-1, and about 28.3% nesfatin-1 positive neurons contained fluorogold. These results indicate that nesfatinergic neurons in the NAc shell send axonal projections to the VMH. Figure 4D illustrated the position of immunohistochemical figures in the NAc.

### Effect of NAc stimulation on VMH GD-responsive neurons

**Figure 5:**
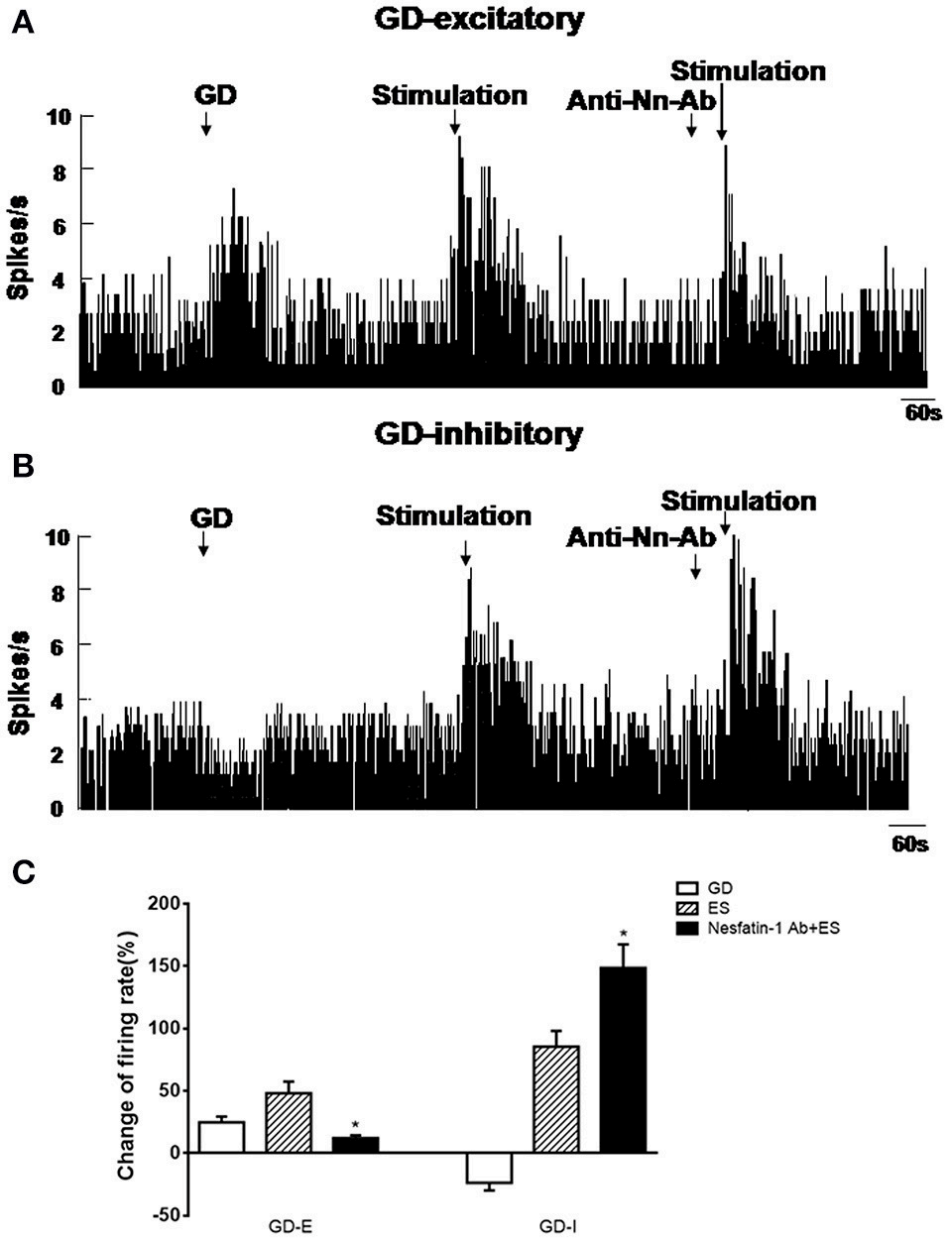
**Effect of NAc electrical stimulation on the firing rate of GD-responsive neurons in the VMH (***n*** = 80). (A)** GD-E neurons in the VMH were mostly excited by NAc electrical stimulation, **(B)** GD-I neurons in the VMH were mostly excited by NAc electrical stimulation. **(C)** Shows the change in firing rate (%) of GD-responsive neurons in the VMH induced by NAc electrical stimulation. Pre-treatment with NUCB2/nesfatin-1 antibody in the VMH reduced the effect of NAc electrical stimulation on VMH GD-responsive neurons. ^*^P < 0.05 vs. electrical stimulation.

Next, we examined the physiological effects of NAc shell modulation of VMH GD-repsonsive neurons. We applied a 20 μA current to NAc shell while simultaneously recording single unit discharges from GD-responsive neurons in the VMH. One hundred and three GD-responsive neurons (54 GD-E and 49 GD-I) in the VMH of 80 rats were found. NAc shell electrical stimulation alone further excited 32 of 54 GD-E neurons (3.73 ± 0.97 to 5.53 ± 0.97 Hz, *P* < 0.05, Figures 5A,C). Notably, pretreatment with nesfatin-1 antibody in the VMH decreased the firing rate to 4.18 ± 1.02 Hz (*P* < 0.05).

When GD-I neurons were examined, 26 of 49 (53.06%) were excited by NAc shell stimulation (2.74 ± 0.71 to 5.08 ± 1.15 Hz, *p* < 0.05, Figures 5B,C). Unlike GD-E neurons, the firing rate of GD-I neurons was increased from 5.08 ± 1.15 to 6.81 ± 1.13 Hz by the nesfatin-1 antibody (*P* < 0.05). Notably, injection of the nesfatin-1 antibody, alone, to VMH did not significantly modify the firing rate of the GD-responsive neurons (GD-E neurons: 3.34 ± 0.95 vs. 3.73 ± 0.97 Hz; GD-I neurons: 2.86 ± 0.91 vs. 2.74 ± 0.71 Hz, *p* > 0.05, Figures 5A,B).

### Effect of the NAc on food intake and gastric functions

**Figure 6:**
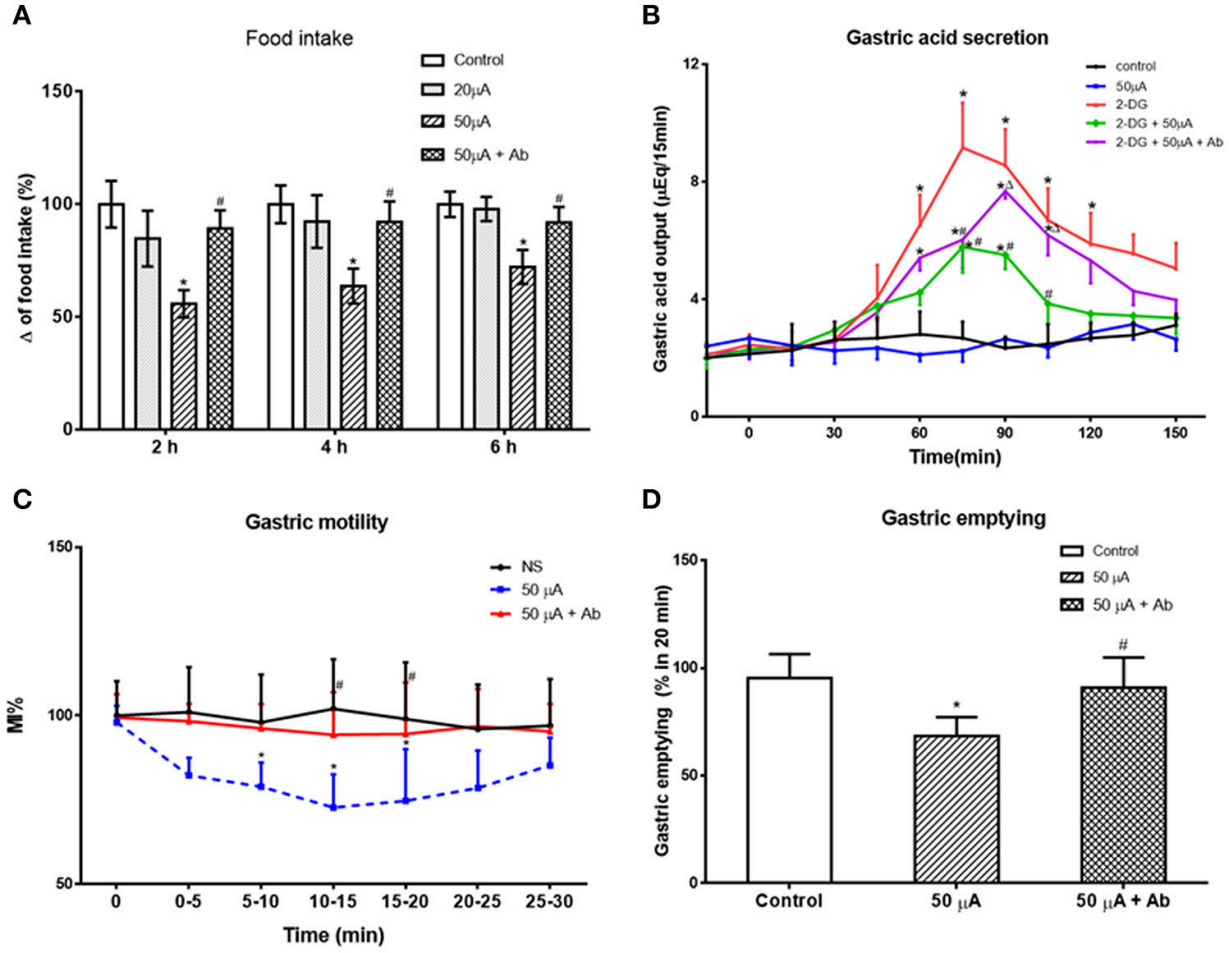
**Effect of electrical stimulation (ES) in NAc shell on food intake and gastric function. (A)** high current ES in NAc inhibited food intake, which was reversed by antibody pretreatment in VMH. ^*^P < 0.05 vs. Control, ^#^*P* < 0.05 vs. 50 μA. **(B)** ES in NAc decreased gastric acid secretion induced by 2-DG, which was partially blocked by a nesfatin-1 antibody. ^*^P < 0.05 vs. Control, ^#^*P* < 0.05 vs. 2-DG, ^Δ^*P* < 0.05 vs. 2-DG + 50 μA. **(C)** Gastric motility was decreased by ES in NAc, ^*^P < 0.05 vs. Control, ^#^*P* < 0.05 vs. 50 μA. **(D)** ES in NAc delayed gastric motility, ^*^P < 0.05 vs. Control, ^#^*P* < 0.05 vs. 50 μA.

Next, we examined the effect of Nac stimulation on food intake and gastric functions, in awake, behaving rats. Low (20 μA) current stimulation to NAc shell had no statistically significant effect on cumulative food intake (*P* > 0.05, vs. control group). High (50 μA) current stimulation decreased cumulative food intake at 2, 4, and 6 h to 55.88 ± 6.11, 63.73 ± 6.76, and 72.19 ± 7.56% of control, respectively (*P* < 0.05, vs. control group, Figure 6A). Interestingly, pretreatment with an anti-nesfatin-1 antibody in the VMH reversed the decrease of food intake induced by NAc stimulation (*P* < 0.05, vs. high current group, Figure 6A).

Fifty microampere current stimulation to NAc shell inhibited 2-DG-induced gastric acid output, reducing gastric acid output 15 min following 2-DG from 9.16 ± 1.53 to 5.52 ± 0.88 μEq (*P* < 0.05). This effect was partially blocked by administration of a nesfatin-1 antibody to VMH (7.68 ± 0.98 μEq at 15 min, *P* < 0.05 vs. 2-DG + 50 μA group, Figure 6B).

Examining the effect of NAc stimulation on gastric motility, MI% was reduced 72.25 ± 9.82% by 50 μA NAc shell stimulation (*P* < 0.05, vs. control group). This effect was partially blocked by a nesfatin-1 antibody, where MI% in the 50 μA + nesfatin-1 antibody group was only 94.32 ± 12.61% of control (*P* < 0.05, vs. 50 μA group, Figure 6C).

Gastric emptying was also affected by 50 μA NAc shell stimulation, where NAc stimulation reduced emptying of the non-caloric viscous solution by 68.36 ± 8.79% (*P* < 0.05, vs. control group). Nesfatin-1 antibody pretreatment partially blocked this effect, as gastric emptying in the 50 μA + nesfatin-1 antibody group was 90.91 ± 14.02% control (*P* < 0.05, vs. 50 μA current group, Figure 6D). These results illustrate that involvement of nesfatin-1 on NAc modulation of gastric functions via projections to the VMH.

## Discussion

**Figure 7:**
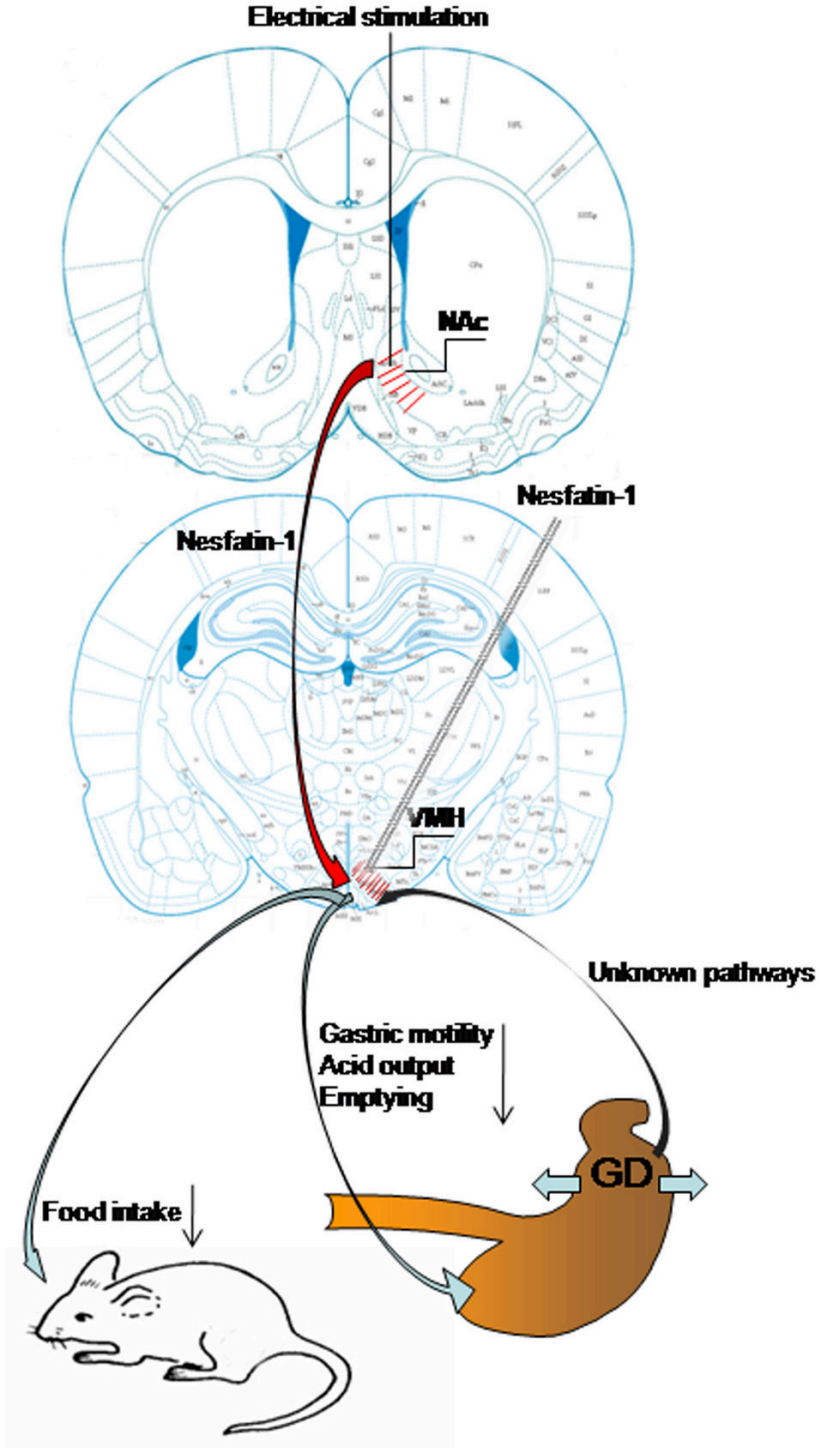
**Schematic of the results of this study**. Gastric distension activates VMH neurons, including nesfatin-1-positive neurons. VMH injection of nesfatin-1 inhibited food intake, gastric motility, gastric acid output, and emptying. Electrical stimulation of NAc modulates VMH neurons and gastric functions via nesfatinergic projection from the NAc to VMH.

In this study, we have demonstrated that GD activates neurons in VMH, including some nesfatin-1-positive neurons. Nesfatin-1 modifies the firing rate of GD-responsive VMH neurons and VMH injection of nesfatin-1 inhibited food intake, gastric motility, gastric acid output, and emptying. We have also shown that electrical stimulation of the NAc affected VMH neurons and gastric functions via nesfatinergic projections from the NAc to the VMH. Figure 7 is a sketch of the results of the present study.

The abundant expression of nesfatin-1 in the VMH is in line with that reported by others (Goebel et al., 2009). In this study, we provide further evidence of the role for nesfatin-1 in VMH neurons by showing its role in GD-induced changes in VMH. Gastric distension, mimicking the rate of gastric emptying of a liquid meal, stimulates neural systems, and induces negative feedback regulation of food intake (Min et al., 2011). Bonnet et al. had reported that NUCB2/nesfatin-1 neurons of the nucleus tractus solitarius (NTS) were sensitive to GD and might contribute to this satiety signal (Bonnet et al., 2013). Our group has shown that nesfatin-1 injection in PVN and ARC also negatively modulate gastric motility (Li et al., 2013; Guo et al., 2015). Therefore, the activation of nesfatin-1 neurons in the VMH is a possible mechanism of GD-induced negative feedback on appetite and food intake.

The predominant physiological effects of nesfatin-1 we observed were an increase of firing rate in GD-E neurons and a reduction of firing rate in GD-I neurons in VMH. These modulatory effects likely enhance the GD-induced satiety signal produced by hypothalamus. Thus, these results support a role for nesfatin-1 in inhibiting food intake as proposed previously (Xu et al., 2013; Guo et al., 2015). Previous work has noted a role for the melanocortin-3/4 receptor (Oh et al., 2006), the oxytocin receptor (Maejima et al., 2009), POMC and CART neurons (Shimizu et al., 2009), or Ca^2+^ signaling pathway (Xia et al., 2012) in nesfatin-1's mechanism of action. In this study, the antagonist of melanocortin-3/4 receptor, SHU9119, inhibited the effect of nesfatin-1 on VMH neurons. Whether a unique, high-affinity, receptor for nesfatin-1 is expressed in brain, or alternatively whether nesfatin-1 exerts its effects through multiple receptors is not known. More studies on nesfatin-1 pharmacology are needed to fully determine its role in satiety.

We also show that delivery of nesfatin-1 to VMH suppresses food intake, gastric acid secretion, gastric motility, and emptying. Previous work has shown that administration of nesfatin-1 to PVN and ARC, or to the whole brain through i.c.v. injection, can also decrease gastric motility in rodents (Xia et al., 2012; Bonnet et al., 2013; Li et al., 2013; Wang et al., 2014). Watanabe et al. reported that intravenous treatment of nesfatin-1 reduced gastric contractions and inhibited cyclical inter-digestive migrating contractions in fasted dogs (Watanabe et al., 2015). These studies show that central or peripheral delivery of nesfatin-1 can negatively regulate gastric motility. Our results indicate that not only gastric motility, but also gastric acid secretion and emptying were inhibited by nesfatin-1, when delivered to the VMH. These functions are similar to leptin, which inhibits food intake, delays gastric emptying and decreases ghrelin expression (Yarandi et al., 2011). Additionally, two studies have found polymorphisms in NUCB2, the gene for the nesfatin-1 precursor protein, associated with obesity (Zegers et al., 2011, 2012). Another line of research has shown acute oral metformin treatment delays gastric emptying, reduces food intake through a leptin-independent mechanism, and activated many nesfatinergic neurons, suggesting a role for nesfatin-1 in metformin's mechanisms of action (Rouquet et al., 2014). Taken together, these data support targeting nesfatin-1 signaling as a leptin-independent strategy for treating obesity.

Our results show that NAc shell express nesfatin-1, and that those nesfatinergic neurons directly project to VMH. The NAc is a key brain nucleus involved in reward processing, motivated behaviors, and regulation of food intake (Kelley et al., 2002). Van der Plasse et al. found that NAc shell stimulation caused increased food intake in rats (van der Plasse et al., 2012). In NAc shell, stimulation of GABA, mu/delta opioid or AMPA receptors alter the feeding state of the animal (Stratford and Kelley, 1997; Stratford et al., 1998; Duva et al., 2005; Castro and Berridge, 2014). Supporting the role for NAc in hypothalamic control of feeding, Duva et al. illustrated anatomical evidence for projections between NAc shell and hypothalamus (Duva et al., 2005). Previous work has illustrated that these connections use primary amino acid neurotransmitters and orexin, and are involved in regulating the hedonic value of tastes, as well as food intake, overall (Urstadt and Stanley, 2015). In this study, we illustrate for the first time a nesfatinergic pathway between NAc shell and VMH. We further show the functionality of this pathway, as pretreatment with a nesfatin-1 antibody in the VMH modified the effect of NAc shell electrical stimulation on GD-responsive VMH neurons. Thus, our result suggest an important role for nesfatin-1 in this NAc shell to VMH connection. Our food intake and gastric function studies illustrate that these nesfatinergic NAc shell to VMH connections are physiologically important. Taken together, these studies confirm the involvement of NAc shell in the regulation of food intake, which likely involves nesfatin-1 signaling in VMH. A better understanding of NAc-hypothalamic connections and nesfatin-1 signaling may be useful in seeking more effective treatments for obesity and other disorders of food intake regulation.

## Conclusions

Nesfatin-1 in the VMH inhibited food intake and gastric functions. A nesfatinergic pathway from NAc shell to VMH was characterized and also shown to be involved in these effects. These results illustrate a novel connection between the reward system and metabolic regulating nuclei in the hypothalamus. Further studies are needed to further characterize this potentially important pathway in the regulation of appetite and gastric functions.

## Author contributions

LX and SG were responsive for the conception, design, and revision of the article. FG, SG, XS, and YG acquired the data. YG and NZ undertook the statistical analysis and interpretation of data, and SG and FG wrote the first draft of the manuscript. All authors contributed to and have approved the final manuscript.

## Funding

This study was supported by the National Natural Science Foundation of China (Grant Number: 81470815, 81270460, 81300281, and 81500414), the Research Award Fund for Outstanding Middle-aged and Young Scientist of Shandong Province (Grant Number: BS2014YY009), the Qingdao Municipal Science and Technology Commission (Grand Number: 14-2-3-3-nsh).

### Conflict of interest statement

The authors declare that the research was conducted in the absence of any commercial or financial relationships that could be construed as a potential conflict of interest.
